# Do coaches and athletes share the same weight-loss practices and perceptions? Insights from 23 combat sport teams

**DOI:** 10.3389/fnut.2026.1802696

**Published:** 2026-03-18

**Authors:** Fanjie Meng, Zhao Zhang, Carl Langan-Evans, Nemanja Lakicevic, Anthony Weldon, Yuming Zhong, Di Wu

**Affiliations:** 1 Department of Martial Arts and Traditional National Sports, Henan Sport University, Kaifeng, Henan, China; 2 Krirk University, Bangkok, Thailand; 3 School of Sport and Exercise Sciences, Liverpool John Moores University, Liverpool, United Kingdom; 4 Faculty of Psychology, Lomonosov Moscow State University, Moscow, Russia; 5 Federal Scientific Center of Psychological and Interdisciplinary Research, Moscow, Russia; 6 Centre for Life and Sport Sciences, Birmingham City University, Birmingham, United Kingdom; 7 Aston Villa Foundation, Aston Villa Football Club, Birmingham, United Kingdom; 8 School of Athletic Performance, Shanghai University of Sport, Shanghai, China; 9 School of Coaching, Shanghai University of Sport, Shanghai, China; 10 College of Professional Tennis, Wuhan City Polytechnic, Wuhan, China

**Keywords:** coaching, sports nutrition, weight class, weight cutting, weight cycling

## Abstract

**Background:**

The present study aimed to examine whether coaches’ weight loss (WL) guidance practices and perceptions are consistent with those of the combat sport (CS) athletes they coach.

**Methods:**

This study employed an observational cross-sectional approach using a survey to ascertain the WL guidance practices and perceptions of CS coaches and WL practices and perceptions of CS athletes and adopted purposeful sampling. In total, 23 coaches and 396 athletes from 23 CS teams who met the inclusion criteria completed the questionnaire. Linear mixed models and generalized estimating equations were used to account for the nested coach–athlete structure.

**Results:**

Significant role differences were observed for habitual WL% and highest WL%, with athletes reporting greater WL magnitudes than coaches recommended. Athletes also reported shorter WL durations than those indicated by coaches. However, after accounting for sex and sport discipline, these role differences were attenuated, indicating that discrepancies were context-dependent rather than uniform across groups. No significant role differences were found for age began WL, perceived coach influence, or perceptions of WL effects on health, performance, and fairness, suggesting substantial perceptual alignment between coaches and athletes. Among 16 WL methods, a significant role difference was identified only for sauna use; this difference was conditioned by sex.

**Conclusion:**

Coaches and athletes demonstrated strong alignment in their perceptions of WL and coach influence. While discrepancies in reported WL practices were observed, these differences appeared to be contextually shaped by sex and sport discipline rather than reflecting a universal athlete–coach divide.

## Background

Systematic reduction of body mass (BM) [i.e., weight-loss (WL)] (1), before competition is a widespread practice in combat sports (CS) and has been extensively documented in the literature (2–4). A recent systematic review indicates that CS athletes typically reduce their BM by less than 5% in the 7–14 days before competition (2). However, athletes in Sambo, MMA, and Muay Thai often undergo more aggressive WL (habitual WL% ranged from 5.2 to 13.0%) (2, 5). Among the most used WL methods are increasing exercise and training with plastic suits, with extreme practices such as using diuretic and diet pills also being reported (2). Despite substantial evidence highlighting the negative impacts of WL on health and performance (6–9), many athletes continue to engage in WL (10–12), illustrating the persistent gap between scientific research and practice.

Among the various individuals who influence athletes’ WL practices, coaches are consistently identified as being the most influential (2). This likely stems from the coach’s overarching role in an athlete’s training, competition preparation, and health management (13, 14). A recent study found that almost all (96%) coaches required their athletes to lose BM and provided guidance through the WL process (15). Additionally, given that many athletes begin pre-competition WL during adolescence (2, 16) and are often lacking sufficient nutritional knowledge (especially sport-specific nutrition knowledge) (17), coaches’ personal experiences with WL therefore become a primary source of WL guidance (13). Certain WL methods, such as increasing exercise volume or training with plastic suits, are inherently tied to training sessions, which may further reinforce the coach’s influence on athletes’ WL practices.

To date, a limited number of studies have explored the coaches’ WL guidance practices and perceptions in CS (15, 18–23). A 1994 study on high school wrestling coaches found they actively influenced athletes’ WL, with 96% assisting in planning and 82% reminding athletes, and even 2% providing diuretics and 1% providing laxatives (18). A 2019 study on judo and taekwondo coaches also revealed that 90% of coaches supervised their athletes’ WL processes (24). On average, judo and taekwondo coaches recommended a WL duration of 16.2 ± 8.2 days, with an average WL magnitude of 1.5 ± 0.7 kg (24). Regarding WL methods, most judo and taekwondo coaches recommended that their athletes practice gradual dieting (92%) (24). A recent study on Chinese CS coaches has revealed longer recommended WL duration (41 days), higher recommended WL% (6.2% of body mass), and distinct preferences for WL strategies—with increased exercise being the most favored approach (57%) (15). These studies affirm that coaches actively supervise WL and provide insight into the construction of attitudes toward their WL guidance practices and perceptions.

However, it has been reported that coaches often lack sufficient knowledge in nutrition and physiology (25, 26), and their primary sources of information are frequently internet or peer discussions (27, 28). Previous research has shown that coaches’ nutritional knowledge can indirectly affect the nutritional and WL practices of elite athletes in weight-class sport (26). Therefore, the WL guidance practices and perceptions of CS coaches may also transfer to the practices and perceptions of the athletes they supervise (16). Although previous studies have examined athletes’ WL practices and coaches’ WL guidance practices (2, 13, 15, 29–31), the participants were not derived from the same sports teams, and thus no direct affiliation existed between the two groups. Consequently, the existing evidence cannot determine whether coaches’ WL guidance and perceptions are truly accepted and applied by their athletes. This question is critical because current findings only indicate that athletes perceive coaches as the most influential figures in their WL behaviors, but this alone does not demonstrate whether coaches’ guidance is indeed effective, nor does it clarify which specific aspects of guidance or perception have an impact. Therefore, this study examined whether coaches’ WL guidance practices and perceptions are consistent with those of the athletes they coach.

## Materials and methods

### Experimental approach to the problem

This study employed an observational cross-sectional approach, using a survey tool to ascertain the WL guidance practices and perceptions of both CS coaches and athletes. To target the relevant population, this study adopted purposeful sampling (13). The study followed the cross-sectional reporting guidelines outlined by the Strengthening the Reporting of Observational Studies in Epidemiology (STROBE) (32), ensuring transparency and consistency in observational research practices.

### Participants

The methods for participant recruitment and questionnaire administration employed in this study were adapted from previous associated studies (13, 15, 29–31). Paper questionnaires were distributed for offline recruitment at a Chinese major national training center in Zhengzhou, Henan. The CS team managers assisted in recruiting participants (coaches and athletes who are affiliated with the same sports team) during daily meetings. All coaches and athletes who agreed to participate in the study were gathered in a conference room to complete the questionnaire. If any questions were unclear, researchers (YZ and ZZ) were available to provide detailed explanations, but they were instructed to avoid suggesting or influencing any specific responses. Before the application of the questionnaire, participants were orally briefed on the instrument for the offline survey. Informed consent was obtained from all participants, including parents or guardians for junior athletes before the questionnaire session. The inclusion criteria for coaches were: (i) working as a CS coach in the past year, directly supervising athletes’ training and competition, with no prerequisite of specific license, qualification, or membership, and (ii) his/her athlete(s) participated in this study and meets the inclusion criteria. The inclusion criteria for athletes were: (i) participation in at least one official CS competition in the last 12 months, (ii) currently training as an athlete on a CS team, and (iii) his/her coach participated in this study and meets the inclusion criteria. In total, 23 coaches and 396 athletes from 23 CS teams (9 sanda, 5 wrestling, 3 boxing, 3 taekwondo, and 3 kickboxing) who met the inclusion criteria completed the questionnaire. Approval for conducting the study was secured from the Ethics Committee of the Shanghai University of Sport (Approval no.: 102772023RT170).

### Questionnaire development and administration

The coach WL guidance questionnaire used in this study was previously used in Zhong et al.’s coach WL guidance questionnaire (15). During Zhong et al.’s validation process (15), content validity of the Chinese version was evaluated by a researcher with extensive CS experience and five CS coaches via pilot tests. This pilot feedback led to some key modifications of questions adapted from Berkovich et al.’s study (24). Additionally, slight modifications were made to the Chinese wording of certain questions based on feedback to better suit the target audience. This study added a question to the survey which focused on the WL% that coaches usually recommend. The survey consisted of 25 questions and featured four sections: (i) general information, (ii) personal experience, (iii) WL guidance practice, and (iv) perception about WL (see Supplementary File 1).

For the assessment of athletes’ WL practices, this study used the questionnaire previously employed by Zhong et al. in their survey of Chinese female adolescent CS athletes (30). The questionnaire consisted of 30 questions and featured four sections: (i) general information, (ii) competition experience, (iii) WL history, and (iv) perception about WL (see Supplementary File 2). Data collection was conducted from August 09 to October 15, 2025. Responses from paper questionnaires were manually input into the WJX web platform (WENJUANXING, www.wjx.cn) by the lead and second author (FM and ZZ).

### Statistical analyses

All analyses were performed using SPSS 27.0 (IBM Corp., Armonk, New York). Descriptive statistics were used to summarize all results. Continuous variables were presented as mean (± SD), and categorical variables as frequencies (%). All WL magnitude reported in this study are expressed as percentage points (0–100%), and all related estimates, standard errors, and confidence intervals are scaled accordingly. Because athletes were nested within coaches, we accounted for the non-independence of observations by using either linear mixed models (LMM) for continuous variables or generalized estimating equations (GEE) for categorical variables, with Coach ID specified as the clustering variable in both approaches. For the LMM analyses, model residuals were examined for normality using Q–Q plots, histograms, and the Shapiro–Wilk test. The residuals were approximately normally distributed, and no transformation of the dependent variables was deemed necessary. For continuous dependent variables (habitual WL%, highest WL%, and age began WL), LMMs were fitted with coach–athlete role (coach vs. athlete), and random intercepts for coaches. Models were estimated using restricted maximum likelihood (REML). Given the hierarchical structure of the data (athletes nested within coaches), multilevel models were initially specified to account for potential between-coach variance. The intraclass correlation coefficients (ICCs) were calculated to quantify the proportion of variance attributable to the coach level. For habitual WL%, between-coach variance was negligible (ICC ≈ 0), indicating minimal clustering effects. To evaluate the robustness of the findings, a sensitivity analysis using SPSS Complex Samples was conducted to obtain cluster-adjusted standard errors under a non-hierarchical modeling framework. Results were substantively consistent with those derived from the multilevel models. Given the relatively small number of clusters (*n* = 23 coaches), a post-hoc power estimation accounting for cluster-level effects was performed. The estimation incorporated the number of clusters, average cluster size, and observed ICC values to assess inferential sensitivity at the cluster level. For categorical dependent variables, GEE models were employed with an exchangeable working correlation matrix and robust standard errors. Multinomial GEE models (generalized logit link) were used for unordered outcomes (e.g., WL duration, perception of coaches’ influence on WL, perceptions of WL effects on health, performance, and fairness, and use of 16 WL methods), while binary GEE models (logit link) were used for dichotomous variables (e.g., use of specific WL methods). For outcomes showing a significant main effect of coach–athlete role, further analyses were conducted to examine potential role × moderator interactions, with sex and sport type included as covariates. Only outcomes with a significant role effect were subjected to these interaction analyses, as the primary aim was to assess whether the role differences were influenced by these moderators. For outcomes with significant role × moderator interactions, simple effect analyses were performed to quantify role differences within each level of the moderator. To reduce the risk of type I error from multiple testing of the 16 individual WL methods, *p*-values from the corresponding GEE models were adjusted using the Bonferroni adjustments. Significance was accepted at *p* < 0.05. The frequency of choice of “*always*” and “*sometimes*” in recommended WL methods is combined to describe the participants’ primary WL methods.

## Results

### General information

The 23 CS teams represented five CS modalities, including sanda (9 coaches and 170 athletes), kickboxing (3 coaches and 81 athletes) taekwondo (3 coaches and 70 athletes), wrestling (5 coaches and 47 athletes), and boxing (3 coaches and 28 athletes), totaling 23 coaches and 396 athletes.

The average age of the coaches (21 males and 2 females) was 30.7 ± 6.7 years old (male 30.0 ± 6.3 years old, female 38.0 ± 6.0 years old). Most coaches held a Chinese primary-level coaching certification (level-1) (*n* = 10, 43%), followed by no coaching certification (*n* = 7, 30%), intermediate-level coaching certification (level-2) (*n* = 3, 13%), advanced-level coaching certification (level-3) (*n* = 2, 9%), and national-level coaching certification (level-4) (*n* = 1, 4%). These coaches began training as athletes in their current sports at an average age of 13.8 ± 1.8 years old, started coaching in the same sport at 21.3 ± 2.8, and had an average of 9.4 ± 6.4 years of coaching experience.

The prevalence of pre-competition WL among the athletes were as follows: sanda (*n* = 130, 76%), boxing (*n* = 19, 68%), wrestling (*n* = 31, 66%), kickboxing (*n* = 53, 65%), taekwondo (*n* = 34, 49%). General information of athletes who engaged in WL (*N* = 267) is presented in Table 1.

**Table 1:** General information (mean and standard deviation) of Chinese CS athletes who engaged in weight loss (*n* = 267).

Variables	All athletes who engaged in weight loss (*n* = 267)			Male athletes who engaged in weight loss (*n* = 208)			Female athletes who engaged in weight loss (*n* = 59)		
	Mean ± SD	Max	Min	Mean ± SD	Max	Min	Mean ± SD	Max	Min
Age (years)	16 ± 2	30	13	16 ± 1.9	25	13	17 ± 3.1	30	13
Body mass (kg)	60.5 ± 11.0	115.0	39.0	61.8 ± 11.5	115.0	39.0	55.9 ± 8.0	76.0	44.0
Stature (cm)	170.8 ± 7.7	193.0	150.0	172.5 ± 7.4	193.0	150.0	164.8 ± 5.4	175.0	150.0
Age began training in current sport (years)	12 ± 2	16	8	12 ± 2	16	10	13 ± 1	16	10
Age began competing in current sport (years)	14 ± 2	19	9	14 ± 2	19	9	15 ± 2	19	12
Off-season body mass (kg)	60.0 ± 11.2	115.0	38.0	61.2 ± 11.6	115.0	38.0	55.9 ± 8.5	77.0	43.0
Competitions participated last competitive season (*n*)	1.7 ± 0.8	8	1	1.8 ± 0.9	8	1	1.7 ± 0.6	3	1
Medals gained during last competitive season (*n*)	1 ± 1	8	0	1 ± 1	8	0	1 ± 1	5	0

### Coaches’ WL guidance practices and perceptions

On average coaches considered 14.9 ± 1.6 years old to be the appropriate age for athletes to undertake their first pre-competition WL. If an athlete currently weighs 60 kg, coaches recommend an average habitual WL of 3.8 ± 1.6% and highest WL 6.7 ± 2.7% BM before competition. Typically, most coaches advised athletes to begin WL 22 + days before the competition (*n* = 21, 91%), while only a small number recommend a duration of 15 to 21 days (*n* = 2, 9%). The frequency of coaches’ recommendations for different WL methods is shown in Table 2. Most coaches perceived themselves as having some influence (*n* = 15, 65%) over their athletes’ WL practices, followed by responses of unsure (*n* = 3, 13%), little influence (*n* = 3, 13%), high influence (*n* = 2, 9%), and no influence (*n* = 0, 0%).

**Table 2:** Frequency analysis (%) of the weight loss methods recommended by coaches (*n* = 23).

Methods	Always		Sometimes		Almost never		Never used		Do not use any more	
	*n*	%	*n*	%	*n*	%	*n*	%	*n*	%
Increased exercise	15	65%	6	26%	0	0%	2	9%	0	0%
Training in plastic suits	10	43%	10	43%	3	13%	0	0%	0	0%
Training in a heated room	6	26%	13	57%	0	0%	4	17%	0	0%
Sauna	3	13%	12	52%	2	9%	5	22%	1	4%
Gradual dieting	7	30%	7	30%	4	17%	3	13%	2	9%
Hot water immersion	0	0%	10	43%	2	9%	10	43%	1	4%
Hot saltwater immersion	0	0%	7	30%	0	0%	15	65%	1	4%
Restricting fluid ingestion	2	9%	4	17%	8	35%	7	30%	2	9%
Use plastic suit all-day	2	9%	2	9%	7	30%	11	48%	1	4%
Skipping meals	0	0%	4	17%	10	43%	8	35%	1	4%
Vomiting	0	0%	1	4%	2	9%	20	87%	0	0%
Fasting	0	0%	0	0%	4	17%	18	78%	1	4%
Spitting	0	0%	0	0%	7	30%	15	65%	1	4%
Laxatives	0	0%	0	0%	2	9%	21	91%	0	0%
Diuretics	0	0%	0	0%	2	9%	21	91%	0	0%
Diet pills	0	0%	0	0%	2	9%	21	91%	0	0%
Others	0	0%	0	0%	0	0%	23	100%	0	0%

The primary reason coaches required their athlete to lose BM was to (i) “compete against lighter opponents to enhance winning potential” (*n* = 20, 87%), followed by (ii) “optimizing athletic performance (e.g., faster speed)” (*n* = 14, 61%), (iii) “athletes weighing above their usual body mass, which may be detrimental to competition performance” (*n* = 14, 61%), (iv) “considering WL as a necessary pre-competition process” (*n* = 2, 9%), (v) “uncertainty but believing it may be beneficial” (*n* = 1, 4%), and (vi) “since everyone cuts body mass, athletes have no choice but to follow” (*n* = 1, 4%).

Half of the coaches believed that WL has no effect on health (*n* = 12, 52%), followed by those who believed it negatively impacts health (*n* = 9, 39%), or improves health (*n* = 2, 9%). Most coaches believed that WL optimizes performance (*n* = 10, 43%), followed by those who considered it having no impact performance (*n* = 7, 30%) or detrimental (*n* = 6, 26%). Regarding fairness in competition, most coaches believed that WL does not create an unfair competition (*n* = 20, 87%), followed by those who were uncertain (*n* = 3, 13%), while no athletes believed it does create unfairness.

### Athletes’ WL practices and perceptions

The average age athletes began WL was 14.7 ± 1.4 years old. Their average habitual and highest WL was 7.3% ± 4.1 and 9.4% ± 4.0% of BM. Athletes usually began to lose weight 15 + days before the competition (64%), followed by 11–14 days (21%), 8–10 days (5%), 6–7 days (4%), 4–5 days (4%), 1–3 days (1%). Most athletes perceived coaches as having high influence (*n* = 99, 37%), over their WL practices, followed by responses of some influence (*n* = 69, 26%), no influence (*n* = 58, 22%), little influence (*n* = 22, 8%), and unsure (*n* = 3, 7%). The WL methods used by athletes are shown in Table 3.

**Table 3:** Frequency analysis (%) of the weight loss methods used by athletes (*n =* 267).

Sources	Always		Sometimes		Almost never		Never used		Do not use any more	
	*n*	%	*n*	%	*n*	%	*n*	%	*n*	%
Increased exercise	133	50%	79	30%	17	6%	31	12%	7	3%
Training in plastic suits	101	38%	78	29%	27	10%	52	19%	9	3%
Skipping meals	44	16%	103	39%	39	15%	70	26%	11	4%
Gradual dieting	44	16%	92	34%	45	17%	69	26%	17	6%
Restricting fluid ingestion	52	19%	83	31%	37	14%	87	33%	8	3%
Training in a heated room	64	24%	45	17%	53	20%	94	35%	11	4%
Spitting	22	8%	39	15%	29	11%	160	60%	17	6%
Sauna	12	4%	48	18%	37	14%	158	59%	12	4%
Vomiting	13	5%	33	12%	21	8%	185	69%	15	6%
Use plastic suit all-day	19	7%	23	9%	57	21%	155	58%	13	5%
Fasting	12	4%	23	9%	56	21%	161	60%	15	6%
Hot water immersion	9	3%	27	10%	27	10%	193	72%	11	4%
Laxatives	4	1%	16	6%	10	4%	223	84%	14	5%
Others	17	6%	2	1%	11	4%	216	81%	21	8%
Diet pills	2	1%	9	3%	8	3%	235	88%	13	5%
Hot saltwater immersion	3	1%	9	3%	20	7%	221	83%	14	5%
Diuretics	3	1%	2	1%	10	4%	238	89%	14	5%

The primary reason reported for engaging in WL was to compete against lighter opponents to increase the chances of winning (*n* = 190, 71%), followed by optimizing athletic performance (*n* = 167, 63%), being above usual weight before a competition (*n* = 102, 38%), “because everyone else is cutting weight, so I have to do it too” (*n* = 10, 4%), “my coach told me to lose weight, so I have to do it” (*n* = 4, 2%), and others (*n* = 3, 1%).

Almost half of the athletes believed that WL has no effect on health (*n* = 125, 47%), followed by those who believed it negatively impacts health (*n* = 83, 31%), or improves health (*n* = 59, 22%). Athletes believed that WL optimizes performance (*n* = 109, 41%), followed by those who considered it having no impact (*n* = 103, 39%) or detrimental (*n* = 55, 20%). Regarding fairness in competition, most athletes believed that WL does not create an unfair competition (*n* = 208, 78%), followed by those who were uncertain (*n* = 50, 19%) and those who believed it does (*n* = 9, 3%).

### The difference of WL practices and perceptions between coaches and athletes

**Figure 1:**
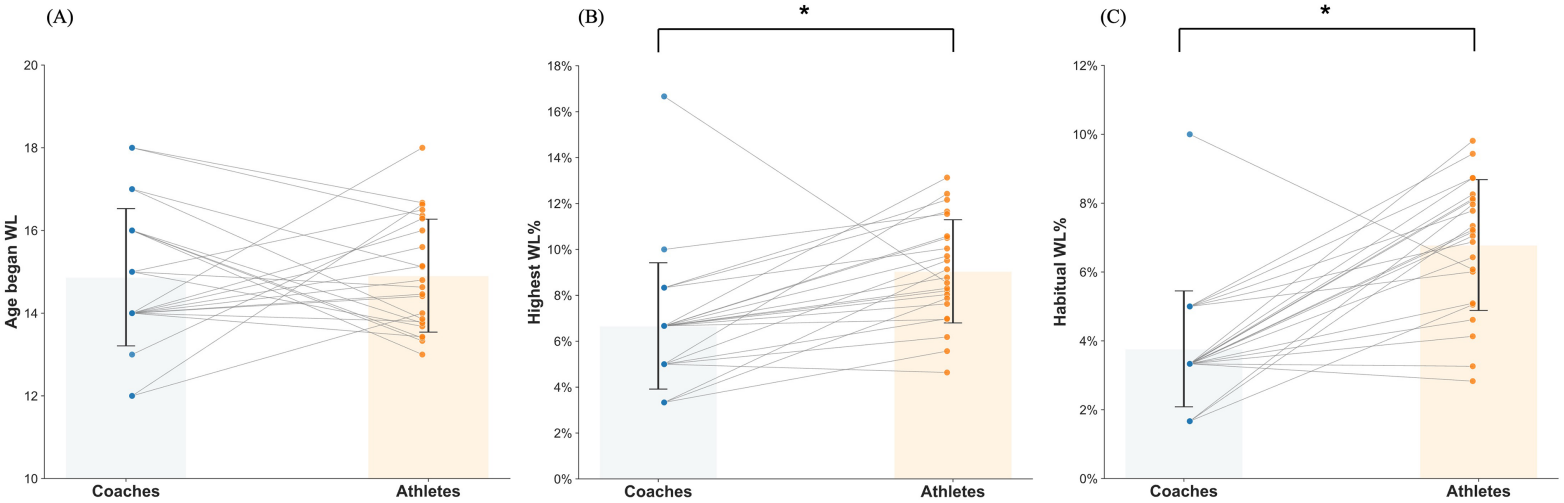
Linear mixed models’ analysis between coaches’ recommendations and athletes’ practice on weight loss variables. WL, weight loss; *, significant difference *p* < 0.05. The connection of two points by a line indicates that they are the coach and athlete of the same sports team. **(A)** Coaches’ recommendations on age began WL vs. athletes’ age began WL. **(B)** Coaches’ recommendations on highest WL% vs. athletes’ highest WL%. **(C)** Coaches’ recommendations on habitual WL% vs. athletes’ habitual WL%.

For habitual WL%, the LMM revealed a significant effect of role (athlete vs. coach), *F*(1, 44.646) = 33.426, *p* < 0.001. Athletes had a significantly higher average habitual WL% than coaches by 3.1% (B = 3.1, SE = 0.5, *t* = 5.782, *p* < 0.001, 95% CI [2.0, 4.2]). Random effects showed essentially zero variance between coaches (*σ*^2^ = 0.000) and zero covariance between athlete and coach measurements, indicating negligible and independent random differences. The ICC was calculated as < 0.001, indicating that virtually no variance in habitual WL% was attributable to between-coach differences, with variance occurring almost entirely at the individual level. Given the negligible clustering effect (ICC < 0.001), the multilevel specification was retained primarily to preserve consistency across models and to reflect the theoretically nested structure of the data (athletes within coaches). A sensitivity analysis using a single-level linear regression model with cluster-robust standard errors at the coach level yielded comparable results (B = 0.034, SE = 0.006, *p* < 0.001, 95% CI [0.022, 0.046]). The magnitude and significance of the effect remained consistent with the multilevel model, indicating that the findings were robust to model specification (Figure 1).

When sex was included as a moderator, the main effect of role for habitual WL% became non-significant (B = 0.019, SE = 0.019, *t* = 1.016, *p* = 0.31, 95% CI [−0.018, 0.055]), indicating that the athlete–coach difference in habitual WL% was no longer statistically detectable. The role × sex interaction was also not significant (B = 0.011, SE = 0.016, *t* = 0.696, *p* = 0.487, 95% CI [−0.021, 0.043]). In contrast, sport discipline significantly moderated the role effect (role × discipline, B = −0.009, SE = 0.003, *t* = −2.693, *p* = 0.011, 95% CI [−0.015, −0.002]). Simple effect analyses by discipline showed significant athlete–coach differences in boxing, sanda, taekwondo, and wrestling, whereas in kickboxing the difference was not statistically significant (B = 0.002, 95% CI [−0.016, 0.020]).

For highest WL%, the LMM revealed a significant effect of role (athlete vs. coach), *F*(1, 47.423) = 10.543, *p* = 0.002. Athletes had a significantly higher average WL magnitude than coaches by 2.4% (B = 2.4, SE = 0.8, *t* = 3.247, *p* = 0.002, 95% CI [0.9, 4.0]). Random effects showed moderate variance between coaches (*σ*^2^ = 0.001) and zero covariance between athlete and coach measurements. The ICC was calculated as 0.500, indicating that 50% of the total variance in highest WL% was attributable to differences between coaches, with the remaining 50% occurring at the individual level. This moderate ICC value supports the use of a multilevel modeling approach to account for the clustering of observations within coaches, as it corrects for the non-independence of nested data and avoids overestimation of statistical significance.

When sex was included as a moderator, the main effect of role became non-significant (B = 0.024, SE = 0.025, *t* = 0.969, *p* = 0.337, 95% CI [−0.026, 0.074]), indicating that the athlete–coach difference in highest WL% was no longer statistically detectable. The role × sex interaction was also not significant (B = 0, SE = 0.022, *t* = 0.013, *p* = 0.990, 95% CI [−0.043, 0.044]). However, a significant interaction between role and discipline was observed (role × discipline, B = −0.010, SE = 0.005, *t* = −2.067, *p* = 0.045, 95% CI [−0.019, 0]), indicating that the magnitude of the athlete–coach discrepancy differed across disciplines. Simple effect analyses revealed significant athlete–coach differences in boxing, sanda, taekwondo, and wrestling, whereas in kickboxing the difference was not statistically significant (B = −0.008, 95% CI [−0.034, 0.018]).

For age began WL, there was no significant effect of role (athlete vs. coach), *F*(1, 43.67) = 0.002, *p* = 0.963, suggesting that athletes’ actual age of starting WL did not differ from the age recommended by coaches. Random effects showed a substantial variance between coaches (*σ*^2^ = 2.22) and negligible covariance between athlete and coach measurements. The ICC was calculated as 0.815, demonstrating that 81.5% of the total variance in age began WL initiation was attributable to inter-coach differences, with only 18.5% of variance occurring at the individual level. This high ICC value provides strong empirical justification for the use of a multilevel modeling framework, which appropriately accounts for the pronounced clustering of observations within coach groups.

For WL duration, the GEE revealed a significant role effect, with athletes reporting shorter durations than coaches (B = −3.961, SE = 0.747, Wald *χ*^2^ = 28.095, df = 1, Bonferroni-adjusted *p* = 0.016, Exp(B) = 0.019, 95% CI [0.004, 0.082]). When sex was included as a moderator, the GEE model incorporating the role × sex interaction did not converge due to numerical instability (Hessian matrix singularity), and parameter estimates could not be reliably obtained. Therefore, this interaction term was not retained in the final interpretation. When sport discipline was included as a moderator, neither the main effect of role (B = −1.486, SE = 2.597, Wald χ^2^ = 0.327, df = 1, *p* = 0.567, Exp(B) = 0.226, 95% CI = [−6.576, 3.605]) nor the role × discipline interaction (B = −0.938, SE = 1.042, Wald χ^2^ = 0.811, df = 1, *p* = 0.368, Exp(B) = 0.391, 95% CI = [−2.980, 1.104]) was statistically significant, indicating that WL duration did not differ between athletes and coaches across sports.

No significant differences were found between athletes and coaches regarding the perceived influence of coaches on WL (B = 0.171, SE = 0.477, Wald χ^2^ = 0.128, df = 1, Bonferroni-adjusted *p* = 1, Exp(B) = 1.186, 95% CI [0.466, 3.022]), nor for perceptions of WL effects on health (B = −0.607, SE = 0.407, Wald χ^2^ = 2.225, df = 1, *p* = 0.136, Exp(B) = 0.545, 95% CI [0.245, 1.210]), performance (B = 0.106, SE = 0.457, Wald χ^2^ = 0.054, df = 1, *p* = 0.816, Exp(B) = 1.112, 95% CI [0.454, 2.722]), or fairness (B = 0.165, SE = 0.675, Wald χ^2^ = 0.060, df = 1, *p* = 0.807, Exp(B) = 1.118, 95% CI [0.314, 4.431]). These results indicate that athletes and coaches generally share similar views on the impact of WL.

Regarding the 16 WL methods, significant differences were only found for Sauna (B = 1.511, SE = 0.482, Wald χ^2^ = 9.812, df = 1, Bonferroni-adjusted *p* = 0.032, Exp(B) = 4.531, 95% CI [1.760, 11.662]), with athletes more likely than coaches to report using these methods. For the remaining methods, no significant role effects were observed (all Bonferroni-adjusted *p* > 0.05), indicating similar use between athletes and coaches. When sex was included as a moderator, the main effect of role was not statistically significant (B = −0.228, SE = 1.190, Wald χ^2^ = 0.037, df = 1, *p* = 0.848, Exp(B) = 0.796, 95% CI = [0.077, 8.205]). However, a significant role × sex interaction was observed (B = 1.652, SE = 0.690, Wald χ^2^ = 5.740, df = 1, *p* = 0.017, Exp(B) = 5.218, 95% CI = [0.301, 3.004]), indicating that the difference between athletes and coaches varied by sex. Simple effect analyses showed that the role difference was significant in both males (B = 1.256, SE = 0.511, *p* = 0.014) and females (B = 4.105, SE = 1.718, p = 0.017). Notably, the magnitude of the role difference was substantially greater among females than males. When sport type was included as a moderator, neither the main effects (B = 5.328, SE = 16.252, Wald χ^2^ = 0.107, df = 1, *p* = 0.743, Exp(B) = 206.021, 95% CI = [−26.526, 37.182]) nor the role × sport interaction (B = −0.582, SE = 2.674, Wald χ^2^ = 0.047, df = 1, *p* = 0.828, Exp(B) = 0.559, 95% CI = [−5.823, 4.659]) were statistically significant.

Overall, these findings suggest that while athletes and coaches differ in the duration of WL practices and in the use of a few specific methods, they generally hold similar perceptions regarding the influence of coaches and the effects of WL on health, performance, and fairness.

## Discussion

This study is the first to examine the alignment between coaches and athletes from the same teams regarding both the practice and perception of WL. The findings revealed that coaches’ perceptions of WL were aligned with those of their athletes, yet notable discrepancies emerged in their reported WL practices. Specifically, significant differences were observed between coaches and athletes in the habitual WL%, highest WL%, allocated WL duration, and the frequency of sauna use; however, after accounting for sex or sport discipline as covariates, the role differences for habitual WL%, highest WL%, WL duration, and sauna use were no longer statistically significant. This pattern indicates that the apparent athlete–coach discrepancies were not uniformly distributed, but were instead contingent upon contextual factors. These results suggest that while coaches and athletes share a similar perception of WL, discrepancies in practice do emerge; however, these discrepancies appear to be contextual rather than universal, shaped by sex and sport-specific environments rather than by role alone.

Coaches and the athletes under their supervision demonstrate consistent perceptions of WL with respect to health, performance, and fairness. This alignment may reflect similarities in WL-related understanding that develop through routine coach–athlete interactions. A majority of coaches and athletes perceive that WL exerts no adverse effect on health, enhances athletic performance, and does not undermine competition fairness. Notably, more than half of the coaches and athletes reported that WL either has no impact on competitive performance or may be detrimental to it. This finding stands in contrast to their reason for WL implementation in both coaches and athletes - to improve the competition performance. This apparent contradiction can be elucidated through multiple aspects. First, if an athlete elects not to engage in WL, they may face a heightened likelihood of competing against taller and heavier opponents, thereby placing themselves at a relative competitive disadvantage. Second, a substantial number of CS athletes perceive WL as “an integral component of training” and a coping mechanism to mitigate pre-competition anxiety (10, 33). Furthermore, many athletes and coaches frame weigh-ins as a “fight before the fight” (10); successful achievement of the target weight (i.e., “winning” on the scale) can augment their sense of accomplishment and increase the perceived probability of succeeding in the actual competition (34). This phenomenon may explain why WL is often described by participants as “horrible but worth it (35).” Nevertheless, it is imperative to recognize that WL is deeply entrenched in the cultural fabric of CS. Research focusing on youth and adolescent CS athletes has documented a similarly high prevalence of WL practices, which mirrors the patterns observed in adult counterparts (16). Given the substantial influence of coaches on athletes’ WL perceptions, it is of paramount importance to provide educational interventions for coaches and athletes regarding the potential adverse effects of WL — effects that can impair both athletic performance and health, and may further culminate in chronic health consequences following the cessation of athletic careers (36).

Coaches and their athletes shared a consistent perception regarding the coach’s influence on athletes’ WL practices. Most coach–athlete pairs within the same team perceived that coaches exerted a moderate influence on athletes’ WL behaviors. Previous research has consistently shown that athletes tend to identify coaches as the most influential figures in shaping WL behaviors (2); however, little was known about whether coaches themselves shared this perception. Our study extends this understanding by demonstrating that coaches’ self-perceived influence aligns with their athletes’ perception of that influence. It is worth noting that although the descriptive results appeared to indicate slight differences between coaches and athletes, this discrepancy arose from aggregated reporting across all participants, whereas the inferential analysis accounted for the nested structure of coach–athlete dyads. This alignment between coaches’ self-perception and athletes’ perception of influence may be understood as reflecting consistency between coaches’ expressed attitudes and athletes’ observed coaching behaviors. Coaches who believe they play a crucial role in their athletes’ WL process are more likely to exhibit behaviors consistent with that belief—such as providing frequent monitoring, explicit feedback, and motivational communication during WL phases. These behaviors make their influence more visible and salient to athletes, reinforcing the athletes’ perception of strong coach involvement. Conversely, coaches who perceive themselves as having limited influence may engage less actively in WL-related guidance, resulting in athletes perceiving lower levels of coach involvement. In this way, the congruence between coaches’ and athletes’ perceptions may be associated with repeated daily interactions, during which coaches’ expressed beliefs and observable behaviors become salient to athletes (37). This finding indicates that coaches’ perceptions of their own importance in athletes’ WL processes are closely associated with athletes’ reported perceptions and behavioral patterns (38). Furthermore, in practical settings, coaches should be encouraged to enhance their awareness of their own influence through reflection and educational initiatives, and to convey WL-related concepts in a more scientifically rigorous and health-focused manner during communication and guidance (16). In essence, enhancing coaches’ awareness and promoting evidence-based guidance may represent a practical strategy for supporting safer WL practices among athletes (3).

Coaches’ perceptions of the appropriate age to initiate WL were closely aligned with the actual age at which athletes began WL. This alignment is readily explicable: in the present study, most coaches had requested their athletes to engage in WL, and when coaches mandated WL for competition preparation, the majority of athletes were likely to comply with this advice—given that WL is commonly perceived as a necessary pre-competition process by CS athletes (10, 33). Prior research has further indicated that adolescent CS athletes appear to be more susceptible to coach influence than their adult counterparts (30). Consequently, the age of athletes’ WL initiation appears to be closely aligned with the timing of coaches’ WL recommendations. Notably, although adolescence constitutes a critical phase of growth and development—during which adequate nutrient intake is essential, particularly for athlete populations—the present study and previous research have documented that most CS athletes initiate WL during adolescence (2, 13, 29–31). This paradox appears unavoidable, as adolescence is when athletes typically begin participating in competitive events. In China, CS teams are generally categorized by competition age groups into adolescent teams (typically municipal-level teams) and adult teams (typically provincial-level teams). If an athlete fails to achieve outstanding competition achievements in adolescent competitions, they will be ineligible for selection to adult teams and thus unable to participate in official adult CS competitions. Therefore, early WL initiation among CS athletes may reflect structural features of the competitive system rather than purely individual decision-making, because WL and competition achievements not only determine athletes’ eligibility to continue participating in CS but also shape their future career trajectories. Moreover, WL appeals to all CS athletes striving for best competitive performance and achievements.

Post-hoc power estimation accounting for cluster-level design effects indicated differential inferential sensitivity across outcomes. For habitual WL%, the negligible ICC (<0.001) resulted in an effective sample size of approximately 412, yielding statistical power exceeding 99% to detect the observed role difference. For highest WL%, the moderate ICC (0.50) reduced the effective sample size to approximately 46, corresponding to an estimated power of ~78%, which remains within acceptable limits for detecting medium-sized effects. In contrast, for age began WL, the high ICC (0.815) reduced the effective sample size to approximately 29, resulting in very low statistical power (~5%) for detecting small between-role differences. Therefore, the non-significant role effect observed for age began WL should be interpreted cautiously, as limited cluster-level power may have constrained inferential sensitivity rather than indicating true equivalence. Importantly, this limitation pertains specifically to small between-role differences and does not undermine the overall pattern of perceptual alignment observed in the study. Outcomes analyzed using GEE were less sensitive to cluster-level variance due to population-averaged estimation; however, Bonferroni adjustments may have further reduced statistical sensitivity. Accordingly, non-significant findings, particularly for high-ICC indicators, should be interpreted with caution.

Coaches and athletes demonstrated broad consistency in the frequency of use for most WL methods, indicating a shared understanding of commonly adopted strategies. However, this apparent alignment was not entirely uniform. Sauna use, for example, revealed a sex-conditioned discrepancy: although the overall role difference was attenuated after adjusting for sex, the significant role-by-sex interaction suggests that the magnitude of athlete–coach divergence differed between males and females. This suggests that certain WL behaviors may be particularly sensitive to sex-specific norms, physiological considerations, or pressures, rather than reflecting a general athlete–coach divide. A similar pattern of conditional discrepancy emerged for WL magnitude. Athletes initially appeared to report higher habitual and highest WL% than coaches recommended, but this divergence was not consistent across all subgroups. Sex largely accounted for the overall difference, whereas sport discipline shaped where the discrepancy persisted. Specifically, athlete–coach differences in WL% were evident in boxing, sanda, taekwondo, and wrestling, but not in kickboxing. This indicates that the gap between recommendation and execution is not a universal behavioral tendency; rather, it appears to be activated within particular sport-specific cultures and competitive structures. In other words, WL practices appear to be shaped not only by role, but also by the normative environment of each discipline. For WL duration, the initial athlete–coach difference was no longer evident after accounting for discipline, suggesting that variation in time allocated to weight reduction is more strongly associated with sport structure than with role-based behavioral divergence. This finding reinforces the idea that some perceived discrepancies between guidance and practice may actually reflect contextual demands rather than intentional deviation.

Taken together, these findings refine the notion of “perceptual consistency but executive discrepancy.” Athletes and coaches generally share similar views on WL, yet divergence in execution emerges under specific contextual conditions. From a behavioral standpoint, this pattern aligns with frameworks such as the Theory of Planned Behavior (39, 40), which posits that shared attitudes and subjective norms do not necessarily translate into identical behaviors when perceived behavioral control or situational constraints differ. This also reflects a practical challenge: even if coaches possess correct WL perceptions and guidance, athletes may still adopt strategies that differ from recommendations in the absence of monitoring or individualized support (37, 41). This finding further emphasizes the importance of enhancing communication and feedback mechanisms between coaches and athletes at the practical level, particularly during the development and implementation of WL plans (42). Future educational initiatives and interventions should not only focus on conveying evidence-based WL concepts but also ensure that these concepts are effectively translated into practical actions.

### Limitations

This study has several methodological limitations. First, the reliance on retrospective self-reported data may have introduced recall bias and social desirability bias, particularly in reporting WL magnitude, duration, and perceptions of coach influence. Athletes completed questionnaires within their team environment, which may have affected their willingness to report extreme practices or critically evaluate coaching guidance, even though responses were anonymous. Although anonymity was emphasized to reduce response pressure, it cannot fully eliminate normative or hierarchical influences within coach–athlete relationships. In addition, the absence of qualitative verification methods (e.g., interviews or focus groups) limits the depth of contextual interpretation and the ability to triangulate reported behaviors. Second, the study did not conduct an *a priori* sample size calculation. Although post-hoc power analysis indicated adequate statistical power for most role comparisons, cluster-level power was limited for outcomes with high intraclass correlation, which may have constrained sensitivity in detecting small between-role differences. From a sampling perspective, the study was conducted within a single training center (the largest CS training base in China), which may limit the generalizability of findings to other regions or organizational systems. Although the center encompasses athletes and coaches across multiple competitive levels and disciplines, the practices and perceptions observed may reflect characteristics specific to this regional training environment. Therefore, caution is warranted when extrapolating these findings to other institutional or cultural contexts. Moreover, while multiple CS disciplines were represented, the number of coaches within each discipline was relatively small, resulting in an imbalance between coaches and athletes across sports. The primary analyses therefore focused on overall role-based differences within the shared institutional context rather than discipline-specific contrasts. Given the limited cluster sizes within individual disciplines, stratified or cross-level interaction analyses would have produced unstable estimates. Accordingly, the findings should be interpreted as aggregated patterns within this training system rather than discipline-specific effects. Future research with larger and more balanced samples across sport types is warranted to explore potential discipline-level heterogeneity.

## Conclusion

Coaches’ perceptions of WL were generally aligned with those of their athletes, yet notable discrepancies emerged in their reported WL practices. Statistically significant differences were observed between coaches and athletes in the habitual WL%, highest WL%, allocated WL duration, and the frequency of sauna use. These results indicate that while coaches and athletes share a similar perception of WL, athletes may not fully adhere to their coaches’ WL guidance in practice, often engaging in shorter WL durations and achieving higher WL% than those recommended by their coaches. Additionally, coaches and their athletes demonstrated consistent perception regarding the coach’s influence on athletes’ WL practices. Overall, these findings highlight patterns of perceptual alignment coexisting with context-dependent practical divergence in WL behaviors, providing descriptive insight into coach–athlete interactions rather than implying causal or prescriptive recommendations.

## Data Availability

The original contributions presented in the study are included in the article/Supplementary material, further inquiries can be directed to the corresponding author.

## References

[1] Zhong Y Tang W Weldon A Gough LA Gee TI Lakicevic N . Reevaluating the definition of rapid weight loss in sports: a call for change. J Int Soc Sports Nutr. (2025) 22:2550312. doi: 10.1080/15502783.2025.2550312, 40849677 PMC12377160

[2] Zhong Y Song Y Artioli GG Gee TI French DN Zheng H . The practice of weight loss in combat sports athletes: a systematic review. Nutrients. (2024) 16:1050. doi: 10.3390/nu16071050, 38613083 PMC11013344

[3] Burke LM Slater GJ Matthews JJ Langan-Evans C Horswill CA. ACSM expert consensus statement on weight loss in weight-category sports. Curr Sports Med Rep. (2021) 20:199–217. doi: 10.1249/JSR.0000000000000831, 33790193

[4] Ricci AA Cassandra E Charles S Peacock CA French DN Stout JR . International society of sports nutrition position stand: nutrition and weight cut strategies for mixed martial arts and other combat sports. J Int Soc Sport Nutr. (2025) 22:2467909. doi: 10.1080/15502783.2025.2467909 PMC11894756 40059405

[5] Barley OR Chapman DW Abbiss CR. Weight loss strategies in combat sports and concerning habits in mixed martial arts. Int J Sports Physiol Perform. (2018) 13:933–9. doi: 10.1123/ijspp.2017-0715, 29283792

[6] Degoutte F Jouanel P Bègue RJ Colombier M Lac G Pequignot JM . Food restriction, performance, biochemical, psychological, and endocrine changes in judo athletes. Int J Sports Med. (2006) 27:9–18. doi: 10.1055/s-2005-837505, 16388436

[7] Franchini E Brito CJ Artioli GG. Weight loss in combat sports: physiological, psychological and performance effects. J Int Soc Sports Nutr. (2012) 9:52. doi: 10.1186/1550-2783-9-52, 23237303 PMC3607973

[8] Gann JJ Tinsley GM La Bounty PM. Weight cycling: prevalence, strategies, and effects on combat athletes. Strength Cond J. (2015) 37:105–11. doi: 10.1519/SSC.0000000000000168

[9] Štangar M Štangar A Shtyrba V Cigić B Benedik E. Rapid weight loss among elite-level judo athletes: methods and nutrition in relation to competition performance. J Int Soc Sports Nutr. (2022) 19:380–96. doi: 10.1080/15502783.2022.2099231, 35859622 PMC9291696

[10] Pettersson S Ekström MP Berg CM. Practices of weight regulation among elite athletes in combat sports: a matter of mental advantage? J Athl Train. (2013) 48:99–108. doi: 10.4085/1062-6050-48.1.04, 23672331 PMC3554040

[11] Pélissier L Ennequin G Bagot S Pereira B Lachèze T Duclos M . Lightest weight-class athletes are at higher risk of weight regain: results from the French-rapid weight loss questionnaire. Phys Sportsmed. (2023) 51:144–52. doi: 10.1080/00913847.2021.2014285, 34875202

[12] Vasconcelos BB Guedes JB Del Vecchio FB. Prevalence, magnitude, and methods of rapid weight loss in national level Wushu Sanda athletes. Sci Sports. (2024) 39:43–50. doi: 10.1016/j.scispo.2022.08.006

[13] Zhong Y Lakicevic N Drid P Gee TI Tang W Sui Y . Prevalence and patterns of pre-competition weight loss practices in Chinese amateur boxers. Int J Sports Sci Coach. (2024) 20:281–90. doi: 10.1177/17479541241295314

[14] Zhong Y Weldon A Bishop C Li Y. Practices of strength and conditioning coaches across Chinese high-performance sports. Int J Sports Sci Sci Coach. (2023) 18:1442–55. doi: 10.1177/17479541231176491

[15] Zhong Y Weldon A Luo Y Kirk C Li P Zhang Z . Hey athlete, you need to cut weight: weight loss guidance practices and perceptions of Chinese combat sports coaches. J Int Soc Sport Nutr. (2025) 22:2565385. doi: 10.1080/15502783.2025.2565385, 41020766 PMC12481538

[16] Lakicevic N Matthews JJ Artioli GG Paoli A Roklicer R Trivic T . Patterns of weight cycling in youth Olympic combat sports: a systematic review. J Eat Disord. (2022) 10:75. doi: 10.1186/s40337-022-00595-w, 35614520 PMC9131524

[17] Dunican IC Galpin A Turner M Reale R. Sleep behaviors and nutritional knowledge in amateur and professional combat sport athletes. J Strength Cond Res. (2024) 38:1627–34. doi: 10.1519/JSC.0000000000004846, 38985931

[18] Marquart LF Sobal J. Weight loss beliefs, practices and support systems for high school wrestlers. J Adolesc Health. (1994) 15:410–5. doi: 10.1016/1054-139X(94)90266-6, 7947857

[19] Sossin K Gizis F Marquart LF Sobal J. Nutrition beliefs, attitudes, and resource use of high school wrestling coaches. Int J Sport Nutr. (1997) 7:219–28. doi: 10.1123/ijsn.7.3.219, 9286745

[20] Umoren J Carper D Smith C. Weight management practices and beliefs of junior and senior high school wrestling coaches. J Am Diet Assoc. (2001) 101:A-85. doi: 10.1016/S0002-8223(01)80292-3

[21] Weissinger E Housh TJ Johnson GO. Coaches' attitudes, knowledge, and practices concerning weight loss behaviors in high school wrestling. Pediatr Exerc Sci. (1993) 5:145–50. doi: 10.1123/pes.5.2.145

[22] Griffin J Harris MB. Coaches’ attitudes, knowledge, experiences, and recommendations regarding weight control. Sport Psychol. (1996) 10:180–94. doi: 10.1123/tsp.10.2.180

[23] Berkovich B-E Eliakim A Nemet D Stark AH Sinai T. Rapid weight loss among adolescents participating in competitive judo. Int J Sport Nutr Exerc Metab. (2016) 26:276–84. doi: 10.1123/ijsnem.2015-0196 26479490

[24] Berkovich BE Stark AH Eliakim A Nemet D Sinai T. Rapid weight loss in competitive judo and taekwondo athletes: attitudes and practices of coaches and trainers. Int J Sport Nutr Exerc Metab. (2019) 29:532–8. doi: 10.1123/ijsnem.2018-0367, 30975001

[25] Danaher K Curley T. Nutrition knowledge and practices of varsity coaches at a Canadian university. Can J Diet Pract Res. (2014) 75:210–3. doi: 10.3148/cjdpr-2014-021, 26067076

[26] Sung JY Lee JH Lee KL. Analysis of the diet, weight-loss behavior, and nutritional knowledge of athletes and coaches in weightclass sports: influence of a coach's nutritional knowledge on athletes. J Int Soc Sports Nutr. (2024) 21:2405159. doi: 10.1080/15502783.2024.2405159, 39287144 PMC11409413

[27] Stoszkowski J Collins D. Sources, topics and use of knowledge by coaches. J Sports Sci. (2016) 34:794–802. doi: 10.1080/02640414.2015.1072279 26222481

[28] Couture S Lamarche B Morissette E Provencher V Valois P Goulet C . Evaluation of sports nutrition knowledge and recommendations among high school coaches. Int J Sport Nutr Exerc Metab. (2015) 25:326–34. doi: 10.1123/ijsnem.2014-0195, 25386951

[29] Meng F Zhong Y Zhang Z Ren Z. Victory above all: the weight loss practices and perceptions of Chinese male kickboxers. PeerJ. (2025) 13:e19709 40656947 10.7717/peerj.19709 PMC12255242

[30] Zhong Y Langan-Evans C Weldon A Roberts CJ Gee TI Lakicevic N . Weight loss practices, perceptions, and eating disorders among Chinese female adolescent combat sports athletes. Int J Sports Sci Sci Coach. (2025) In press. doi: 10.1177/17479541251377640

[31] Zhong Y Tang W Gee TI Li M Jiang H Yin M . Weight loss practices in Chinese national and international-level Sanda athletes. J Int Soc Sports Nutr. (2025) 22:2551216. doi: 10.1080/15502783.2025.2551216, 40876444 PMC12395625

[32] von Elm E Altman DG Egger M Pocock SJ Gøtzsche PC Vandenbroucke JP . The strengthening the reporting of observational studies in epidemiology (STROBE) statement: guidelines for reporting observational studies. Int J Surg. (2014) 12:1495–9. doi: 10.1016/j.ijsu.2014.07.013 25046131

[33] Wilbraham SJ Elliott D Miller PK. It's just how we do it: social processes in rapid weight loss for combat sports. Health Psychol Behav Med. (2024) 12:2433517. doi: 10.1080/21642850.2024.2433517, 39624786 PMC11610240

[34] Baribeau V Kirk C Le DQ Bose A Mueller A French D . Rapid weight gain and weight differential predict competitive success in 2100 professional combat-sport athletes. Int J Sports Physiol Perform. (2023) 18:85–94. doi: 10.1123/ijspp.2022-0204, 36473482

[35] Smith KA Naughton RJ Langan-Evans C Lewis K. “Horrible-but worth it”: exploring weight cutting practices, eating behaviors, and experiences of competitive female Taekwon-do athletes. A mixed methods study. J Clin Sport Psychol. (2024) 18:150–64. doi: 10.1123/jcsp.2021-0103

[36] Miles-Chan JL Isacco L. Weight cycling practices in sport: a risk factor for later obesity? Obes Rev. (2021) 22:e13188. doi: 10.1111/obr.13188 33372395

[37] Beckner BN Record RA. Navigating the thin-ideal in an athletic world: influence of coach communication on female athletes' body image and health choices. Health Commun. (2016) 31:364–73. doi: 10.1080/10410236.2014.957998, 26361233

[38] Chartrand TL Bargh JA. The chameleon effect: the perception-behavior link and social interaction. J Pers Soc Psychol. (1999) 76:893–910. doi: 10.1037/0022-3514.76.6.893, 10402679

[39] Ajzen I. The theory of planned behavior. Organ Behav Hum Decis Process. (1991) 50:179–211. doi: 10.1016/0749-5978(91)90020-t

[40] Ajzen I. Perceived behavioral control, self-efficacy, locus of control, and the theory of planned behavior. J Appl Soc Psychol. (2002) 32:665–83. doi: 10.1111/j.1559-1816.2002.tb00236.x

[41] Mountjoy M Ackerman KE Bailey DM Burke LM Constantini N Hackney AC . 2023 International Olympic Committee's (IOC) consensus statement on relative energy deficiency in sport (REDs). Br J Sports Med. (2023) 57:1073–98. doi: 10.1136/bjsports-2023-106994, 37752011

[42] Coker-Cranney A Reel JJ. Coach pressure and disordered eating in female collegiate athletes: is the coach-athlete relationship a mediating factor? J Clin Sport Psychol. (2015) 9:213–31. doi: 10.1123/jcsp.2014-0052

