# Supplementary material for: Do coaches and athletes share the same weight-loss practices and perceptions? Insights from 23 combat sport teams
Source: Front Nutr. 2026 Mar 18;13:1802696. doi: 10.3389/fnut.2026.1802696 (PMC13038610; doi:10.3389/fnut.2026.1802696)
Supplement: Supplementary file 1 [file Table_1.docx]

**格斗项目教练员赛前减重调查**

**尊敬的教练员:**

您好！

我们是上海体育大学HP研究团队，目前正在进行我国格斗运动员赛前减重调查，我们非常迫切地想要了解教练员对运动员减重的看法，诚邀您参与本问卷的填写。

本次调查为匿名，所收集的资料只用于本研究，您不会受到任何的影响，请您根据自己的实际情况真实填写。衷心感谢您的支持与配合!

符合本研究的教练员必须执教过参加比赛的运动员。如有任何疑问，请联系负责人：钟裕明，19121719175

请尽可能认真地回答问题，上海体育大学感谢您的参与！

填答要求：所有问题均需填答。______为填空题，直接往里面填答案；（ ）为选择题，需在对应的正确选择左侧括号打勾，每一选择题右侧会标注是单选题还是多选题。问卷为正反面，一共有2面，请完整作答。

黎涌明

上海体育学院HP研究团队

**1、**我已阅读上面的知情同意书，同意参与本问卷填写。选择“是”即代表签署知情同意书。 （ ）是 （ ）否

请注意：由于这是一份匿名调查，因此您无需签名。如果您完成问卷，则同样视为您同意参与这项研究。

今天的日期： 年 月 日

**2、**年龄：_________岁

**3、**性别：（ ）男，（ ）女

**4、**您带的运动队名称：_________

**5、**您当前执教的运动**项目**？（单选题）

（ ）拳击 （ ）散打 （ ）跆拳道 （ ）摔跤 （ ）柔道 （ ）自由搏击 （ ）巴西柔术 （ ）UFC（ ）综合格斗 MMA

（ ）桑搏 （ ）泰拳 （ ）其它 请说明

**6、**您当前的**最高**级别教练员证书为？（单选题）

（ ）无教练员证书（ ）初级 （ ）中级 （ ）高级 （ ）国家级

**7、**您**几岁开始**参加您当前项目的**训练**？（作为运动员） ______岁（填写年龄，勿写年份）

**8、**您**几岁开始**担任您当前项目的**教练**？ ______岁（填写年龄，勿写年份）

**9、**您当前带的运动员**主要**参加什么级别的比赛（单选题）

（ ）校级比赛（如校运会）

（ ）区或市级比赛（如周口市比赛）

（ ）省级比赛（如河南省比赛）

（ ）国家级比赛（如全国锦标赛）

（ ）国际级比赛（如世界杯、世锦赛、亚运会）

**10、**您是否了解您的运动员们的体重？

（ ）是 （ ）否

**11、**您是否曾让您的运动员进行赛前减重？

（ ）是 （ ）否

**12、**您是否曾经亲自指导您的运动员进行减重（例如告诉他们怎么吃，多跑步）？ （ ）是 （ ）否

**13、**通常情况下，您在运动员减重方面和医务人员或营养师的交流有多频繁？

（ ） 总是 （ ）经常 （ ）有时 （ ）很少 （ ）从未

**14、**您指导运动员减重的依据多大程度依赖以下来源？（每一种方法勾选一种程度）

| 来源 程度 | 极大 | 较大 | 中等 | 较小 | 极小 |
| --- | --- | --- | --- | --- | --- |
| 自己在运动员期间积累的经验 |  |  |  |  |  |
| 教练员培训课程教的内容 |  |  |  |  |  |
| 其它教练的做法和建议 |  |  |  |  |  |
| 营养师和医生的建议 |  |  |  |  |  |
| 网络上的建议（如抖音、微信公众号） |  |  |  |  |  |
| 其他（请说明）：________________ |  |  |  |  |  |

**15、**您认为运动员在**多少岁开始第一次赛前减重**比较合适？ ______岁（填写年龄，勿写年份）

**16、**您的一个运动员当前体重为60kg，您推荐他在比赛前最多减去多少公斤？ ______公斤kg

**17、**您的一个运动员当前体重为60kg，您通常推荐他在比赛前减去多少公斤？ ______公斤kg

**18、**通常情况下，您推荐运动员在比赛前多少天开始进行赛前减重？ ______天

**19、**您认为自己在运动员**赛前减重方面**（不是训练方面）的**影响程度有多大**？

（ ）没有影响 （ ）影响不大 （ ）不确定 （ ）有一些影响力 （ ）非常有影响力

**20、**下表列出了几种减重的方法，请您勾选您对不同方法对推荐程度（每一种方法勾选一种频率）。

| 方法 使用频率 | 总是推荐 | 有时推荐 | 很少，几乎从来没有推荐过 | 以前推荐过，但已不再推荐 | 从来都没推荐过 |
| --- | --- | --- | --- | --- | --- |
| 逐步节食（2周内或更长时间） |  |  |  |  |  |
| 每天不吃1或2餐 |  |  |  |  |  |
| 禁食（整天不吃东西） |  |  |  |  |  |
| 限制液体（如水）摄入 |  |  |  |  |  |
| 增加运动（比平时更多） |  |  |  |  |  |
| 有意地在加热的训练房进行训练 |  |  |  |  |  |
| 蒸桑拿 |  |  |  |  |  |
| 穿橡胶/塑料服（即控体重服）进行训练 |  |  |  |  |  |
| 在不运动的情况下穿控体重服 |  |  |  |  |  |
| 吐口水 |  |  |  |  |  |
| 泻药 |  |  |  |  |  |
| 利尿剂 |  |  |  |  |  |
| 减肥药 |  |  |  |  |  |
| 呕吐 |  |  |  |  |  |
| 热水浴 |  |  |  |  |  |
| 热盐水浴 |  |  |  |  |  |
| 其他方法（请说明）：________________ |  |  |  |  |  |

**21、**您为什么让运动员进行赛前减重？【多选题】

（ ）优化运动员的运动表现，如速度更快，更灵活

（ ）可以与低体重组的运动员竞争，提升获胜可能

（ ）运动员比赛前体重高于平常体重，不利于比赛

（ ）大家都减，所以他们也不得不减

（ ）只是认为赛前减重是比赛前的必要流程

（ ）我也不确定，只是感觉这样会有利于比赛

（ ）我不推荐运动员进行赛前减重

（ ）其他，请说明

**22、**您认为减重对于运动员健康状况的影响是？（单选题）

（ ）提升健康水平 （ ）无影响 （ ）损害健康水平

**23、**您认为赛前减重对运动员比赛时的表现有多重要？

（ ）非常重要 （ ）比较重要 （ ）中等重要 （ ）不太重要 （ ）完全不重要

**24、**您认为减重对于比赛表现的影响是？（单选题）

（ ）优化表现（打得更好） （ ）无影响 （ ）降低表现（打得更差）

**25、**您认为赛前减重，是否会导致比赛的不公平？（单选题）

（ ）是，导致比赛不公平 （ ）否，比赛仍然公平 （ ）不确定

感谢您的回答！
